# Public Health

**Published:** 1897-10-02

**Authors:** 


### Public Health.

In an address delivered at the Sanitary Institute, on September 27th, Dr. Louis Parkes drew attention to the decline which had taken place in the marriage-rate during recent years. The birth-rate also had fallen. The results of improved sanitation were thus much complicated statistically by the altered marriage-rate and birth-rate. It had been contended that our charitable systems and methods interfered with the evolutionary law of the survival of the fittest, and to a certain extent this was true. Famine and plague had in the past tended to produce a strong and virile race. Many people now reached the age of 21 who, under the conditions of life years ago, would have died before attaining that age, but the expectation of life at ages above 26 had diminished in males, and he thought that the shortening of individual life and the steady diminution of the marriage and birth rates were evidences of degeneration.

